# The Primary Folding Defect and Rescue of ΔF508 CFTR Emerge during Translation of the Mutant Domain

**DOI:** 10.1371/journal.pone.0015458

**Published:** 2010-11-30

**Authors:** Hanneke Hoelen, Bertrand Kleizen, Andre Schmidt, John Richardson, Paraskevi Charitou, Philip J. Thomas, Ineke Braakman

**Affiliations:** 1 Department of Chemistry, Faculty of Science, Cellular Protein Chemistry, Bijvoet Center for Biomolecular Research, Utrecht University, Utrecht, The Netherlands; 2 Department of Physiology, University of Texas Southwestern Medical Center, Dallas, Texas, United States of America; University of South Florida College of Medicine, United States of America

## Abstract

In the vast majority of cystic fibrosis (CF) patients, deletion of residue F508 from CFTR is the cause of disease. F508 resides in the first nucleotide binding domain (NBD1) and its absence leads to CFTR misfolding and degradation. We show here that the primary folding defect arises during synthesis, as soon as NBD1 is translated. Introduction of either the I539T or G550E suppressor mutation in NBD1 partially rescues ΔF508 CFTR to the cell surface, but only I539T repaired ΔF508 NBD1. We demonstrated rescue of folding and stability of NBD1 from full-length ΔF508 CFTR expressed in cells to isolated purified domain. The co-translational rescue of ΔF508 NBD1 misfolding in CFTR by I539T advocates this domain as the most important drug target for cystic fibrosis.

## Introduction

The Cystic Fibrosis Transmembrane conductance Regulator (CFTR) is a multi-spanning membrane protein that not only functions as a cAMP-dependent chloride channel but also interacts with other proteins to mediate ion conductance at the cell surface of lung and intestinal epithelial cells [1], [2], [3]. The 1,480 amino acids form five major domains: two membrane-spanning domains (MSD1 and MSD2), two cytosolic nucleotide-binding domains (NBD1 and NBD2), and a unique cytosolic regulatory domain (R-domain) that is not found in other members of the ATP-binding Cassette (ABC) Transporter C class.

More than 1,500 mutations found in the gene encoding CFTR lead to cystic fibrosis, the most common lethal genetic disease amongst Caucasians. The most frequent CF-causing mutant, ΔF508, lacks a phenylalanine in NBD1; it is efficiently retained in the ER [4] and almost completely degraded by the proteasome via ER associated degradation [5], [6].

Structural models of CFTR [7], [8], [9] place F508 at the interface between NBD1 and the 4^th^ intracellular loop (ICL4), located within MSD2. Studies on ΔF508 CFTR folding showed that the side chain loss impaired domain-domain interactions within CFTR [10], and that ΔF508 increased protease susceptibility of NBD2 and MSD1 in a post-translational fashion [11], [12]. On the other hand, *in vitro* the ΔF508 mutation does affect NBD1 folding [10], [13], [14] directly, suggesting that deletion of F508 may induce several folding defects, which eventually cause ER retention and degradation.

ΔF508 CFTR can be rescued from retention in the ER by lowering temperature of cells expressing ΔF508 CFTR [15], by addition of chemical chaperones [16], [17], [18], or by introducing suppressor mutations [19]. Teem and coworkers [19] identified two mutations, G550E and I539T, that both significantly increased plasma membrane levels of ΔF508 CFTR and improved channel activity [19], [20], [21].

We have established a CFTR folding assay that allows analysis of co- and post-translational folding of CFTR. Using limited proteolysis performed directly on newly synthesized radiolabeled nascent chains of increasing lengths, full-length CFTR, and isolated domains, but also on purified NBD1 domain, in parallel with biophysical studies, we explored when and where in the full-length structure ΔF508 CFTR misfolds. We found that ΔF508 CFTR affects both cell biological and biophysical stability of the NBD1 domain, already co-translationally and independent of other domains. Introduction of I539T, but not the G550E suppressor mutation, counteracted all folding defects within NBD1, whereas both mutations rescue CFTR trafficking to the cell surface. As mouse CFTR already has a threonine in the human I539 position [19], this residue may act as natural intragenic, intradomain suppressor and hence may contribute to the somewhat milder nature of lung disease in CF mice [22].

## Results

### Small conformational defect in ΔF508 CFTR

**Figure 1:**
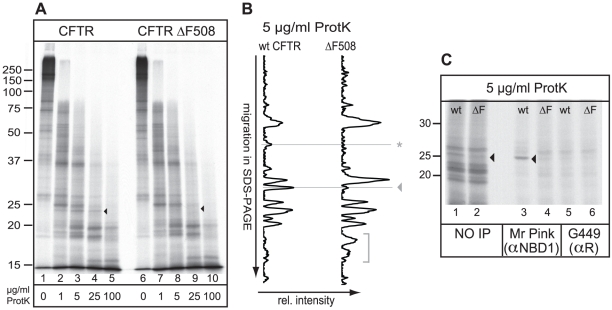
Minimal and local misfolding of ΔF508 CFTR. (A) Both CFTR and ΔF508 CFTR were translated *in vitro* in the presence of ^35^S-methionine and cysteine and semi-permeabilized HT1080 cells for 60 min. Cells containing radiolabeled CFTR proteins were washed, lysed in Triton X-100, and prepared for limited proteolysis using increasing concentrations of proteinase K. The proteolytic digests were analyzed by 12% SDS-PAGE. The conformational difference between wild-type CFTR and ΔF508 CFTR is indicated by an arrowhead. (B) Relative intensities of all protease resistant fragments from a total 5 µg/ml Proteinase K digest, as in Figure 1A, were determined by total lane quantitation (Quantity One software Biorad). The y-axis represents electrophoretic mobility in 12% SDS-PA gel and the x-axis the relative intensity of the protease resistant fragments. The horizontal lines indicate the structural differences as described in A. The horizontal line indicated with an asterisk represents yet unidentified changes in the proteolytic pattern as a result of the ΔF508 mutation. The bracket represents small proteolytic fractions detected in both mutants. (C) Wild-type and ΔF508 CFTR were synthesized as in a, were subjected to 5 µg/ml proteinase K and NBD1-originated fragments were immunoprecipitated with polyclonal antibodies directed against NBD1 (Mr Pink) or against the R-region (G449). Arrowhead marks the NBD1-related fragment.

To determine conformational differences between wild-type and mutant CFTR, we used limited proteolysis of radiolabeled CFTR with a selection of proteases. Wild-type and ΔF508 CFTR were *in vitro* translated and translocated into the ER membrane of digitonin-permeabilized human HT1080 cells in the presence of ^35^S-methionine/cysteine. After 60 min of translation these newly synthesized radiolabeled proteins were solubilized in Triton X-100 and subjected to limited proteolysis using a concentration range of proteinase K to probe their conformation (Figure 1A). This assay is based on the relative protease resistance of folded domains compared to unstructured or misfolded regions [11], [12], [23], [24], [25]. Because CFTR is the only radiolabeled protein in this assay we directly analyze all protease resistant fragments on SDS-PAGE that originate from the complete protein without the caveats of methods requiring immunoprecipitations [24].

Comparing the proteolytic patterns of wild-type and ΔF508 CFTR we found that their protease susceptibility patterns were very similar (Figure 1A). Only a single proteinase K resistant fragment of ∼25 kDa that was present in wild-type CFTR (Figure 1A, lanes 2–4, ◂) was absent in ΔF508 CFTR (Figure 1A, lanes 7–9, ◂). Quantification of the relative intensities of all Proteinase K resistant fragments (5 µg/ml) confirmed this observation (Figure 1B, same symbols as in Figure 1A). In addition a more subtle conformational change in ΔF508 CFTR appeared (Figure 1B, *) and some smaller fragments (Figure 1B,]), suggestive of increased proteolytic susceptibility of the mutant. Because the assay is capable of detecting substantial misfolding of CFTR [24], these results suggest that ΔF508 CFTR had profound conformational perturbations although most of the structure was remarkably similar to that of wild-type CFTR.

### ΔF508 affects CFTR structure locally

To identify the domain origin of the protease resistant fragments we previously used a series of C-terminally truncated CFTR constructs with stop codons behind each subsequent domain [24]. The protease resistant fragment of ∼25 kDa that was lost in ΔF508 CFTR did arise in constructs truncated behind NBD1 or downstream, but not in the shorter construct truncated behind MSD1 (data not shown), implying that this fragment related to NBD1. A polyclonal antibody raised against NBD1, with three prominent epitopes in NBD1, (Mr. Pink) specifically immunoprecipitated the ∼25 kDa fragment from a total pool of protease resistant fragments (Figure 1C, lanes 3 and 4), whereas an antibody against the R-region did not (Figure 1C, lanes 5 and 6). Our results demonstrate that folding mutation ΔF508, which efficiently retains CFTR in the ER, caused misfolding primarily in NBD1, the domain in which it resides.

### ΔF508 destabilizes isolated NBD1 both *in vitro* and *in vivo*

**Figure 2:**
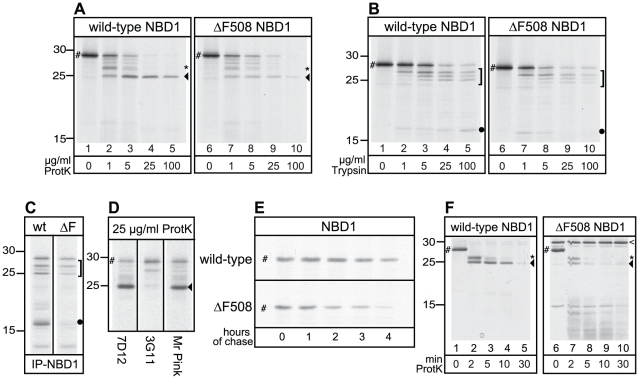
The effect of ΔF508 mutation on NBD1 alone. (A) Wild-type and ΔF508 NBD1 were *in vitro* translated for 30 min, treated with indicated proteinase K concentrations as in Figure 1, and analyzed using 15% SDS-PAGE. The full length NBD1 domain is indicated by “#”, the asterisk (*) indicates the 27 kDa fragment, arrowhead (◂) indicates the 25 kDa fragment. (B) Similar experimental conditions as described in A, but using TPCK-trypsin as protease. A bracket (]) marks the triplet of protease resistant fragments and the dot (•) marks the 17 kDa fragment. (C) Wild-type and ΔF508 NBD1 were synthesized as in A, treated with 100 µg/ml trypsin, and fragments were immunoprecipitated with antibody 7D12 against NBD1. Fragments are labeled similar as in B. (D) Wild-type NBD1 was synthesized as in A, treated with 25 µg/ml proteinase K, and fragments were immunoprecipitated with the 7D12, 3G11 and Mr Pink antibody, recognizing specific epitopes within NBD1. Fragments are labeled similar as in A. (E) CHO cells expressing wild-type or ΔF508 NBD1 were pulse-labeled with ^35^S-methionine and cysteine for 5 min and chased for indicated times. NBD1 was immunoprecipitated using polyclonal antibody Mr Pink and analyzed using 15% SDS-PAGE. NBD1 indicated by “#”. (F) Purified human wild-type and ΔF508 NBD1, indicated by “#”, were incubated with 2 µg/ml Proteinase K for 0, 2, 5, 10 and 30 minutes at room temperature. Proteolytic digests were separated using 15% SDS-PAGE and visualized by silver staining. Asterisk (*) and arrowhead (◂) indicate 27 and 25 kDa fragments resp., and are similar as in A. The open arrowhead (<) indicates a protease resistant background band in the purified ΔF508 NBD1.

To find out whether ΔF508 misfolds NBD1 also in the absence of the other CFTR domains and out of biological context, we analyzed *in vitro* translated wild-type and ΔF508 NBD1 domain in our assays and compared their protease susceptibilities. Wild-type and mutant NBD1 were equally susceptible to proteinase K (Figure 2A, #). With increasing protease concentrations several smaller resistant fragments appeared, which were more rapidly degraded in ΔF508 NBD1 (Figure 2A, marked by symbols). The prominent ∼25 kDa band present in wild-type NBD1 (Figure 2A, ◂) was almost completely digested in ΔF508 NBD1. We found that this band represents an NBD1-related fragment indistinguishable (when analyzed side by side; not shown) from the fragment lost in the digests from full-length ΔF508 CFTR (Figure 1A, ◂). Two other protease resistant fragments showed decreased intensity in ΔF508 NBD1 digests as well: of ∼27 kDa (Figure 2A, *, compare lanes 2 and 7) and ∼17 kDa (only visible in longer exposures, see below).

The trypsin digests showed a similar result: NBD1 was equally susceptible to the protease irrespective of the F508 deletion, but the resulting triplet of protease resistant fragments was again more sensitive in ΔF508 (Figure 2B, lanes 8–10,]) than in wild-type NBD1 (Figure 2B, lanes 3–5). We found the ∼17 kDa protease resistant fragment only in the wild-type NBD1 proteolysis pattern (Figure 2B, •). Immunoprecipitation with an NBD1-specific monoclonal antibody (7D12) (see Materials and Methods) clearly confirmed the NBD1-identity of these fragments and showed that the 17 kDa fragment was absent in the trypsin digest of ΔF508 NBD1 (Figure 2C). Antibody mapping showed that the 25 kDa NBD1 fragment that is protease susceptible in ΔF508 was recognized by antibodies Mr. Pink (directed against various epitopes within NBD1) and 7D12 (epitope 531–540), but not by 3G11 (epitope 396–405) (Figure 2D, ◂), 10B6.2 (epitope 399–408, not shown), or G449 (raised against R-region residues 693–716 but also recognizing C-terminal residues in NBD1, not shown). This suggests that the 25 kDa NBD1 fragment had lost its N- and C-termini and that the NBD1 core structure was destabilized by deletion of F508. Irrespective of protease used, proteolytic fragments of ΔF508 NBD1 were less stable than fragments of wild-type domain. We therefore concluded that deletion of F508 destabilizes isolated NBD1 core domain in biological context as has been shown before *in vitro* [10], [13], [14].

Because full-length CFTR ΔF508 is degraded efficiently by the proteasome and because deletion of F508 altered NBD1 conformation directly, we anticipated that this conformational change would affect biological stability of the NBD1 domain in intact cells. CHO cells were transfected and pulse-labeled to follow NBD1 stability with time (Figure 2E). Wild-type NBD1 was relatively unstable already as only ∼30% of pulse-labeled protein was left after 4 hours of chase, but degradation of ΔF508 NBD1 indeed was significantly faster with 90% of molecules degraded after 4 hours (Figure 2E).

The *in vitro* translation mimics *in vivo* conditions in many important regards: the ER is relatively intact and the reticulocyte lysate used as source of cytosol is full of chaperones and other folding enzymes, which exert their effect on newly synthesized full-length CFTR but also on isolated NBD1. If bound tightly, chaperones and other interacting proteins may leave footprints on the CFTR domains as well by shielding protease cleavage sites. To distinguish the role of cellular context in NBD1 misfolding from a direct effect of the ΔF508 mutation on NBD1, we examined the stability of purified human NBD1 domains in the absence of any other proteins. We used the same limited proteolysis folding assay, but instead fixed the protease concentration and varied the time of digestion at room temperature. We subjected purified wild-type and ΔF508 NBD1 to 2 µg/ml proteinase K and analyzed all fragments on a silver-stained SDS-PA-gel (Figure 2F). The resulting proteolytic pattern was remarkably similar to the pattern of newly synthesized *in vitro* translated NBD1 (Figure 2A). Again the wild-type NBD1 showed a protease resistant fragment of 25 kDa while the corresponding 25 kDa fragment of ΔF508 NBD1 was quickly degraded (Figure 2F, ◂, compare lanes 3&4 with lanes 8&9). The smaller fragments below 15 kDa represent degradation products of the destabilized 25 kDa core domain of ΔF508 NBD1. These fragments are not found in digests of in vitro translated NBD1 because they may lack radiolabeled methionines and cysteines. These results clearly showed that deletion of F508 induced intrinsic misfolding independent of biological conditions and interacting proteins.

**Figure 3:**
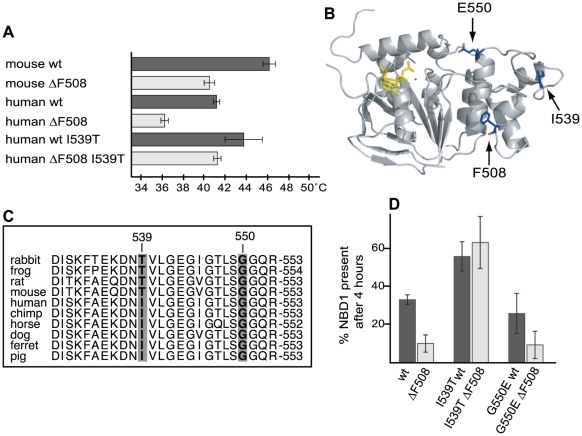
Stability of ΔF508 NBD1 is restored by introducing I539T. (A) Thermal denaturation of NBD1 variants, as indicated in the Figure. Bars represent average (n = 3) melting temperatures (T_M_), error-bars are SD. (B) Crystal structure of mouse NBD1 (1R0W). F508, T539 and G550 are shown in blue, and ATP in yellow. (C) Aligning CFTR sequences of several species revealed that an isoleucine or threonine on position 539 is species dependent. CFTR sequences are from *Oryctolagus cuniculus* (rabbit), *Xenopus laevis* (frog), *Rattus norvergicus* (rat), *Mus musculus* (mouse), *Homo sapiens* (human), *Pan troglodytes* (chimpanzee), *Equus caballus* (horse), *Canis lupus familiaris* (dog), *Mustela putorius furo* (ferret), *Sus scrofa* (pig). (D) CHO cells transiently expressing NBD1 with and without indicated mutations were pulse-labeled for 5 minutes and chased for 4 hours as in Figure 2E. The percentage of labeled NBD1 present after 4 hours of chase was quantified (n = 3, data represented ± SEM).

Application of the limited proteolysis conformational assay to the same protein in purified form and in biological context allowed comparison of conformational differences *in vivo* and *in vitro*. Prior biophysical characterization [10] showed that deletion of F508 affects the yield of purified NBD1 refolding in vitro. To assess the effects of mutations on the stability of the domain we analyzed the thermal stability of the purified NBD1 proteins that were used for limited proteolysis: while wild-type human and mouse NBD1 thermal stability were different, the decrease in melting temperature due to the absence of F508 was similar in both cases (Figure 3A).

### Only I539T but not G550E suppresses the ΔF508 phenotype in NBD1

As the folding defect in ΔF508 CFTR arose in NBD1, we asked whether this defect could be rescued in NBD1 as well. Teem and colleagues identified two suppressor mutations (G550E, I539T) in NBD1 that were located in the same subdomain as F508 (Figure 3B), and that each partially rescued ΔF508 CFTR from the ER [19]. We therefore examined whether the I539T mutation stabilized purified ΔF508 NBD1 (Figure 3A), and found that I539T completely rescued thermal stability of ΔF508 NBD1, and improved stability of wild-type NBD1 as well. Another interesting aspect to residue 539 is that it varies between isoleucine and threonine in different species (Figure 3C), with Ile the residue in man and Thr the residue in mouse, suggesting that mice in principle carry an intragenic suppressor to the ΔF508 mutation. Indeed, thermal stability curves of mouse NBD1s were indistinguishable from those of the corresponding human I539T NBD1s (Figure 3A).

To establish whether the *in vitro* suppression of ΔF508 by I539T leads to improved *in vivo* stability of NBD1, we expressed wild-type and ΔF508 NBD1 with or without suppressor mutation I539T in CHO cells, and measured each protein's half-life (Figure 2E). For comparison we included the G550E suppressor mutation. The two mutations exerted very different effects in that G550E did not affect stability of wild-type or ΔF508 NBD1, while I539T measurably stabilized the domain (Figure 3D). The amount of ΔF508 NBD1 remaining after 4 hours of chase improved from 10% to ∼60% by insertion of I539T, whereas wild-type NBD1 improved from 30% to ∼60%, consistent with the improved melting temperatures we measured. We concluded that the I539T suppressor mutation rescues the thermodynamic and biological stability of ΔF508 NBD1.

### I539T but not G550E fully restores the conformational defect in ΔF508 NBD1

**Figure 4:**
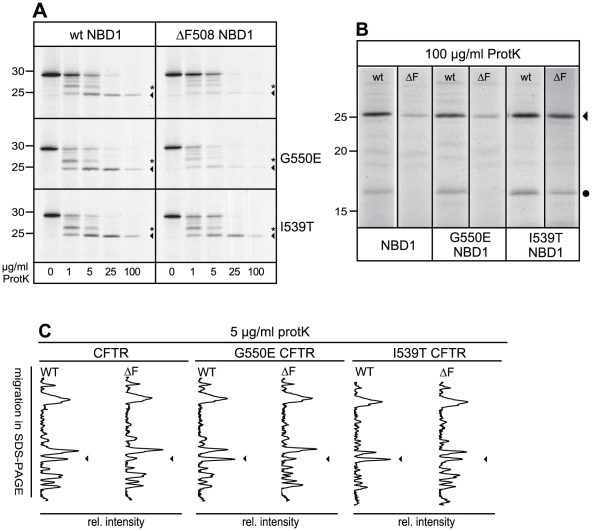
Rescue of NBD1 conformation by the I539T suppressor mutation. (A) Wild-type and ΔF508 NBD1 (top panel) mRNAs containing the G550E (middle panel) or I539T (bottom panel) mutation were *in vitro* translated in the presence of ^35^S-labeled methionine and cysteine and analyzed by 15% SDS-PAGE after proteinase K treatment. Asterisk indicates the 27 kDa fragment, arrowhead indicates the 25 kDa fragment. (B) Longer exposure of the 100 µg/ml proteinase K digest of *in vitro* translated NBD1, from same experiment as shown in B, showing the rescue of the 17 kDa band by the I539T but not by the G550E mutation. Gel lanes are aligned on the 25 kDa bands. (C) CFTR molecules containing the indicated mutations were *in vitro* translated, analyzed using 12% SDS-PAGE and lanes were quantified as described in Figure 1B. The arrowhead indicates the 25 kDa fragment, which has slightly decreased mobility when the I539T mutation is present.

To establish whether the improved stability of the I539T mutant was due to rescued conformation, we *in vitro* translated wild-type and ΔF508 NBD1 with or without suppressor mutations and monitored changes in proteolytic digestion (Figure 4A). Again, I539T, but not G550E, caused a dramatic effect on the proteinase K digest, particularly detectable at 5 µg/ml. Notably, the I539T mutation restored the wild-type NBD1 pattern in ΔF508: both protease resistant bands of ∼25 and 27 kDa that had been lost in ΔF508 NBD1 returned upon mutation of I539 to Threonine (Figure 4A, ◂). Comparing longer exposures of the 100 µg/ml ProtK treatment of the NBD1 molecules revealed that only the I539T mutation restored the ∼17 kDa protease resistant band that had been lost in ΔF508 NBD1, whereas G550E had no measurable impact (Figure 4B, •). Performing similar limited proteolysis experiments on purified ΔF508 NBD1 with or without the I539T mutation confirmed that this suppressor mutation specifically restored protease resistance of the 25 and 17 kDa fragments (data not shown).

To determine whether the rescue of ΔF508 NBD1 occurred also in full-length CFTR, we i*n vitro* translated full-length wild-type and ΔF508 CFTR, with or without the suppressor mutations, and digested each sample with proteinase K. Lane quantitiation of 5 µg/ml proteinase K digests showed recovery of the ∼25 kDa proteinase K-resistant fragment (Figure 4C, ◂). The I539T mutation itself slightly decreased electrophoretic mobility of NBD1 and CFTR (not shown), but also of the 25 kDa fragment, suggesting that this fragment contained the I539T mutation. We concluded that I539T but not G550E rescues the ΔF508 phenotype by completely restoring NBD1 conformation and stability.

**Figure 5:**
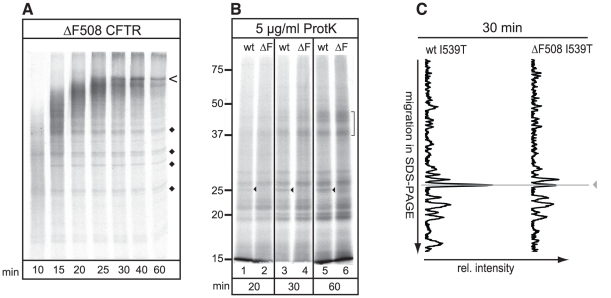
Co-translational misfolding and rescue of NBD1. (A) After 5 min pre-warming the translation mix, we added ^35^S-methionine and followed CFTR synthesis in the SP-cell system for 10–60 min. Analysis using 10% SDS-PAGE directly visualized CFTR nascent chain elongation with time. Full-length ΔF508 CFTR (<) first appeared after 30 min of translation, “⧫” indicates persistent unfinished nascent chains. (B) Wild-type and ΔF508 nascent chains were translated *in vitro* and harvested after 20, 30, or 60 min of synthesis. All nascent chains were subjected to increasing proteinase K concentrations and proteolytic fragments were separated by 12% SDS-PAGE. In the 5 µg/ml proteinase K treatment shown here, the NBD1-related 25 kDa fragment is marked by an arrowhead. The bracket indicates increased protease resistance of CFTR domains as a result of nascent chain elongation. (C) Similar experimental conditions as in B but with the I539T mutation in wild-type and ΔF508 CFTR background. Nascent chains were harvested after 30 min of synthesis and protease resistant fragments were quantified as in Figure 1B. Arrowhead indicates the NBD1-related 25 kDa fragment.

Because NBD1 in wild-type CFTR folds already during synthesis [24], we asked whether NBD1 misfolding and rescue in ΔF508 CFTR occurred during nascent chain elongation and co-translational folding. During the first 30 minutes of *in vitro* translation we monitor only nascent chains and thus co-translational folding of CFTR; after this, full-length CFTR appears [24] (Figure 5A). We harvested nascent chain populations of wild-type and ΔF508 CFTR after 20 and 30 min of synthesis, as well as full-length proteins (60 min) (Figure 5A), and subjected them to treatment with 5 µg/ml of Proteinase K (Figure 5B). Already in the small ΔF508 CFTR nascent chains synthesized within 20 min, the ∼25 kDa NBD1-related fragment was absent, while it was present in wild-type CFTR nascent chains (Figure 5B, cf. lanes 1 and 2). Throughout ongoing synthesis and folding of C-terminal portions of the protein (30 and 60 min), detectable as protease resistant bands with lower electrophoretic mobility (Figure 5B, lanes 3–6,]), the protease sensitive conformation of ΔF508 CFTR remained evident. A similar experiment was done with I539T CFTR and ΔF508 I539T CFTR. Lane quantitation of the proteolytic fragments after 30 minutes of synthesis showed a protease resistant 25 kDa fragment for both wild-type and ΔF508 CFTR containing the additional I539T mutation (Figure 5C). These results show that the NBD1 conformation that leads to protection from limited proteolysis already formed co-translationally, and that ΔF508 CFTR was deficient in this process but was rescued by I539T during synthesis.

Both I539T and G550E partially restore “band C” levels of ΔF508 CFTR. i.e. molecules that obtained complex glycosylation in the Golgi complex [19]. Increased levels of complex glycosylated CFTR can result from increased stability on the plasma membrane (longer half-life) or improved maturation at the ER membrane or both. We found that only the I539T suppressor restored ΔF508 NBD1 domain stability suggesting that multiple mechanisms can contribute to rescue of ΔF508 CFTR. To analyze whether the I539T suppressor improved CFTR maturation like G550E [20], [21] we used a pulse-chase approach to monitor both rate and efficiency of ΔF508 CFTR rescue.

**Figure 6:**
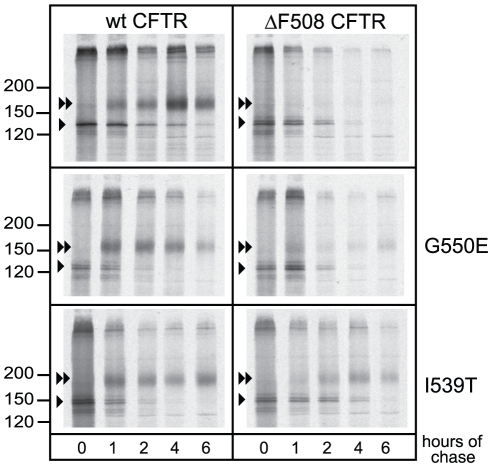
Suppressing the ΔF508 phenotype *in vivo*. HeLa cells transiently expressing CFTR with the indicated mutations were pulse-labeled with ^35^S-methionine and cysteine for 15 minutes and chased for the indicated times. CFTR molecules were immunoprecipitated from radiolabeled lysate using polyclonal antibody directed against the R-domain (G449). Samples were analyzed using 7.5% SDS-PAGE. The arrowhead indicates the ER oligomannose form (B-band) and the double arrowhead indicates complex glycosylated CFTR (C-band).

Transfected HeLa cells were radioactively pulse-labeled to follow maturation of full-length CFTR over time by using the acquisition of complex glycans in the Golgi apparatus as a measure of maturation. While the majority of wild-type CFTR molecules had reached the Golgi complex after 2–4 hours (Figure 6 upper left panel, ◂◂, C-band), ΔF508 CFTR was retained in the ER (Figure 6 upper right panel, ◂, B-band) and degraded by the proteasome. Introducing either G550E or I539T within ΔF508 CFTR partially countered misfolding and enhanced export from ER to Golgi (Figure 6). The I539T suppressor was much more effective than G550E in rescuing ΔF508 CFTR: the majority of CFTR molecules now reached the Golgi complex, whereas some loss of signal still occurred for G550E, implying some residual degradation. On top of this rescue of ΔF508 CFTR, both suppressor mutations also increased maturation rate of wild-type CFTR molecules: they reached the Golgi complex faster and, 2 hours after synthesis, almost no core glycosylated (ER-resident) CFTR was visible anymore. We conclude that, while both mutations rescue full-length CFTR to the plasma membrane, the I539T mutation rescues the ΔF508 phenotype within the isolated NBD1 domain already during its synthesis whereas G550E practically bypasses the folding defect in NBD1 and rescues *via* an alternative mechanism.

## Discussion

The cystic fibrosis ΔF508 mutation that resides in the NBD1 domain of CFTR directly affects stability of this domain in vitro. We here show that mutant NBD1 not only lacks thermal stability but also cell biological stability, as the isolated domain was degraded faster than wild-type NBD1 in intact cells. We found, using limited proteolysis, that ΔF508 CFTR misfolded already co-translationally, as soon as the NBD1 domain had been synthesized. This primary folding defect arose independent of other domains and other cellular components. The I539T suppressor mutation but not G550E in the same subdomain rescued the defect and restored NBD1 conformation to wild-type, pinpointing the subdomain with the mutation as the primary target for therapeutic intervention in cystic fibrosis.

### Co-translational folding defect

We studied the earliest folding events of ΔF508 CFTR at the ER membrane and found that the folding defect induced by deletion of F508 arose co-translationally, immediately upon synthesis of NBD1. Our data suggest that the recognition that leads to degradation of ΔF508 CFTR molecules already occurs at the time of NBD1 synthesis and before downstream domains are being synthesized. This is consistent with the co-translational ubiquitination of CFTR [26], but also with the many chaperones that indeed recognize this ΔF508 NBD1 folding defect, such as Hsc70/Hdj-2, which bind a CFTR MSD1-NBD1 fragment twice as much when F508 is missing [27]. Knockdown of, or displacement from the Hsp90 co-chaperone Aha1 restores plasma membrane levels of ΔF508 CFTR [28], [29]. By keeping CFTR molecules in a soluble state [30] the chaperones suppress aggregation of NBD1 [27], [31] until RMA1 or CHIP are recruited to facilitate co- or post-translational degradation respectively [32], [33]. The NBD1 domain emerges not only as the primary site for misfolding but also as the first domain in ΔF508 CFTR that is recognized and targeted for degradation.

### NBD1 as primary site for misfolding in ΔF508 CFTR

The ΔF508 mutation directly affected the isolated NBD1 domain. NBD1 showed this change not only when purified, but also when expressed in cells, and alone or as part of full-length ΔF508 CFTR, in nascent chains or in C-terminally truncated chains that contained NBD1. Limited proteolysis is based on the relative protease resistance of folded domains compared to unstructured or misfolded regions. In our experiments relative intensities changed rather than the sizes of fragments, which showed that the same proteolytic cleavage sites were used but had become more accessible in mutant protein. This points to a change in stability of NBD1 and not necessarily a large change in conformation.

Our data is consistent with previous in vitro folding studies on isolated wild-type and ΔF508 NBD1 [10], [13], [14], which show that ΔF508 has a measurable effect on the yield with which the isolated NBD1 domain folds. An elaborate mutagenesis analysis showed that the lack of the backbone due to F508 deletion results in this inefficient folding of NBD1 [10] which is in line with our work showing that the primary folding defect of ΔF508 CFTR is within NBD1. However human ΔF508 NBD1 crystal structure was highly similar to the structure of wild-type NBD1 [34], [35], [36]. Yet, to purify and crystallize ΔF508 NBD1 required either suppressor mutations or deletion of flexible loops.

### Where in NBD1 is the defect?

Proteolytic digests of purified NBD1 revealed two major protease resistant fragments, one of ∼25 kDa and a second smaller fragment of ∼17 kDa. The 25 kDa fragment represents the bulk of the “core domain” of NBD1 lacking both N- and C-termini, and the 17 kDa fragment is a part of this. The same core domain was found in the crystal structure of human NBD1 [34]. Antibodies recognizing the core of the NBD1 sequence or the entire domain (660, Mr Pink & 7D12) specifically recognized both fragments, while antibodies mapped to the N-terminal epitopes of NBD1 (10B6. 2 & 3G11) or the far C-terminus (G449) did not. The N- and C-termini of NBD1 thus were removed first by the proteases, independent of the ΔF508 mutation, which explains why wild-type and mutant NBD1 disappeared at similar protease concentrations and incubation times. These initial protease cleavage sites likely reside in RI and RE as they are mobile [37], and the N-terminal strand may stay associated until denaturation by SDS, although the core domain does not need RI and RE for stability [36]. Both 25 kDa and 17 kDa fragments did show increased protease susceptibility in the ΔF508 background, which was rescued completely by the I539T suppressor mutation, not only in terms of protease susceptibility but also its *in vivo* stability and function.

In full-length CFTR we detected the ∼25 kDa core NBD1 fragment but not the ∼17 kDa fragment, most likely because of its weaker radiolabeling (considering the lower number of methionines and cysteines in this fragment), or because of the overlap in gel with other fragments deriving from other CFTR domains.

### Reverting the ΔF508 phenotype

While the I539T mutation rescued NBD1, G550E hardly affected the isolated ΔF508 NBD1 domain. Still, both suppressor mutations rescued full-length CFTR from its retention in the ER and allowed CFTR channel activity at the cell surface [19], [20]. This implies that there is more than one route to rescue, either by correcting the primary defect in NBD1 or by bypassing this primary defect and rescuing domain assembly downstream [11].

After (mostly co-translational) folding of the individual domains [24], these domains need to assemble. Studies on interacting domains [38], [39], [40] and molecular modeling [7], [8], [9] have drawn a clear picture of the domain interactions in CFTR, with the 2 MSDs and the 2 NBDs all 4 interacting with each other, similar to the homologue Sav1866 [41]. In particular the assembly of NBD1 with the 4^th^ intracellular loop of CFTR (ICL4) in MSD2 has received much attention [8] and is likely to be the process immediately downstream of NBD1 and MSD2 domain folding. The F508 deletion was shown to increase protease susceptibility also of the MSD1 and NBD2 domains, suggesting additional important domain-domain interactions for folding [11], [12].

Where G550E does not measurably rescue NBD1 misfolding, it does rescue CFTR functioning and therefore is likely to rescue at least one of the downstream domain assembly steps. The G550E mutation is located in the highly conserved ABC transporter signature motif of NBD1 (LSG^550^GQ) and, according to the Sav1866 homology model [8], is in close proximity to NBD2. The relevance of this is unclear though, as stabilization of the NBD1-NBD2 dimer [42] does not rescue ΔF508 CFTR maturation [43]. While G550E only slightly increases steady state plasma membrane levels of ΔF508 CFTR, it does enhance CFTR mediated chloride currents [19] and restores CFTR channel activity by increasing the duration of channel opening and thereby open probability (P_o_) [20].

The I539T mutation, by contrast, increases chloride channel activity at the plasma membrane (Eric J. Sorscher, Birmingham, personal communication) by increasing the number of CFTR molecules at the cell surface [19] (and Gergely Lukacs, Toronto, personal communication). Complete rescue of NBD1 folding appeared, in spite of the profound effect on CFTR maturation, insufficient for full functional rescue of the CFTR molecule, suggesting that the ΔF508 NBD1 still was hampered in some domain-domain interactions. The crystal structure of NBD1 and the structural model of CFTR provides no obvious hint towards the molecular mechanism of rescue by the I539T mutation.

### Correction of the cystic fibrosis defect

Partial rescue of CFTR release from the ER and (hence) of its function may be sufficient for rescue from CF disease. While estimates vary, an increase of 10% CFTR channel activity on the plasma membrane has been predicted to be the lower limit sufficient to relieve the CF clinical phenotype [44]. Alignments of members of the ABCC (ABC-transporter subfamily of CFTR) revealed that most members of the ABCC family have a 539T suggesting an evolutionary benefit to having a threonine in this position rather than an isoleucine. Mice also have this intragenic suppressor. We here show that the 539 residue completely accounted for the stability difference between mouse and human purified NBD1, and predict that the recently generated CF ferret [45] and CF pig [46] may be better model organisms for some of the clinical phenotypes in cystic fibrosis.

## Materials and Methods

### Cell lines

HT1080 cells (ATCC) were grown in DMEM supplemented with 8% fetal calf serum. The human cervical carcinoma cell line HeLa (ATCC) was grown in MEM supplemented with 10% fetal calf serum and non-essential amino acids and Chinese hamster ovary cells (CHO) were cultured in α-MEM supplemented with 8% fetal calf serum. All cells were cultured in media containing 100 U/ml each of penicillin and streptomycin and 2 mM Glutamax I and kept in humidified incubators at 37°C containing 5% CO_2_. All cell culture media and reagents were obtained from Invitrogen.

### Antibodies and purified protein

G449 [24], [47] polyclonal antisera directed against the CFTR R-region (residues 693–716) were generously provided by Drs Angus Nairn (Rockefeller University, New York NY, USA) and Hugo de Jonge (Erasmus University, Rotterdam, The Netherlands). Mr Pink polyclonal rabbit antiserum was raised by the Braakman lab against purified human NBD1 and characterized to recognize all forms of NBD1, folded, misfolded and denatured. The mouse monoclonal 7D12 was raised by the Thomas lab against purified human NBD1. The same antigen was used by Dr. Bill Balch (Scripps Research Institute, La Jolla CA, USA) who generously provided 3G11 rat monoclonal antibody and by Dr. Eric Sorscher (Gregory Fleming James Cystic Fibrosis Research Center, Birmingham Alabama, USA), who generously provided 10B6.2 mouse monoclonal antibody. All purified NBD1 was supplied by the Thomas lab [48]. Epitope mapping was done by Dr Gergely Lukacs: Mr Pink recognizes various epitopes in NBD1, 7D12 sees epitope 531–540, 3G11 sees epitope 396–405, and 10B6.2 recognizes epitope 399–408). All information concerning protein and antibodies can be found at http://www.cftrfolding.org/reagentrequests.htm.

### Cloning and constructs

The C-terminally truncated hCFTR proteins used in this study were constructed as described before [24]. For transfections hCFTR constructs were subcloned into the pcDNA3 vector using NotI and XhoI (Fermentas). The ΔF508 phenotype was introduced in pBS hCFTR 642X (N-NBD1) and pBS CFTR 837X (N-R) using XbaI and SphI (New England Biolabs). cDNA of hNBD1 (T^389^-G^673^) was cloned by PCR using primers (forward: 5′-GGAATTCCCCGGGGCCACCATGACTACAGAAGTAGTGATGGAG-3′; reverse: 5′-GCGATATCCTATCCTTCTAATGAGAAACGGTG-3′), ligated into pJET vector and subcloned to pcDNA3 vector using EcoRI and EcoRV (Fermentas). The deletion of F508 and the reverting mutations G550E and I539T were introduced both in full- length CFTR and NBD1 by side directed mutagenesis using primers (amino acid change underlined, I539T: 5′-CCAAGTTTGCAGAGAAAGACAAT**ACC**GTTCTTGGAGAAGGTGGAATC-3′ G550E: 5′-GGAGAAGGTGGAATCACACTGAGT**GAG**GGTCAACGAGCAAGAATTTCTTTAGC-3′ ΔF508: 5′-GGCACCATTAAAGAAAATATCATTGGTGTTTCCTATGATGAATATAG-3′) and all constructs were sequence verified.

### Transient expression

HeLa and CHO cells were seeded in 8.5 cm^2^ dishes to reach 50–60% confluency on the day of transfection. To transfer DNA into cells we used a linear 25 kDa polymer polyethylenimine (PEI) dissolved in water to 1 mg/ml, pH adjusted to 7.4, and filtered sterilized (Polysciences). The DNA/PEI complexes were pre-incubated for 15 minutes at room temperature in 150 mM NaCl and added to cells 20 hours before experiments.

### Pulse-chase analysis

Cells were transfected with pcDNA3-CFTR or pcDNA3 -NBD1 constructs containing indicated mutations. The pulse-chase assay was performed as described before [49]. Briefly, HeLa or CHO cells were starved for 15–25 min in MEM without cysteine/methionine (ICN biomedicals) supplemented with Glutamax I (InVitrogen). The HeLa cells expressing full-length CFTR were pulse-labeled for 15 min with ^35^S-methionine and cysteine (60 µCi/dish, Easytag™ Express Protein Labeling Mix, Perkin Elmer) and CHO cells expressing NBD1 were pulse-labeled for 5 min (30 µCi/dish). CFTR was immunoprecipitated from the radiolabeled non-denaturing lysate (20 mM MES, 50 mM Tris-Cl pH 7.4, 100 mM NaCl, 1% Triton X-100, 1 mM PMSF, 10 µg/ml each of chymostatin, leupeptin, antipain, and pepstatin) using G449 antiserum directed against the R-domain, and NBD1 was immunoprecipitated in a similar manner using polyclonal antiserum directed against the NBD1 domain (Mr Pink). The samples were analyzed on SDS-PA gel (7.5% for full-length CFTR, 15% for NBD1), prepared for fluorography, dried, and exposed to film (Kodak Biomax MS).

### *In vitro* translation in the presence of semi-permeabilized cells

This method [50] was adapted to study CFTR nascent chain elongation as described before [24]. In brief, NBD1 or CFTR mRNA was *in vitro* translated in rabbit reticulocyte lysate (Promega) using ^35^S-methionine and cysteine and for translation of full-length CFTR in the presence of freshly prepared semi-permeabilized (SP) HT1080 cells. After incubation at 30°C, the translation was stopped by adding ice-cold KMH buffer (110 mM KOAc, 20 mM HEPES pH 7.2, 2 mM MgOAc) containing 1 mM cycloheximide. The SP-cells were spun down at 4°C, washed with KHM buffer and lysed in KHM containing 1% Triton X-100. Either 2x Laemmli sample buffer was added (final concentrations 200 mM Tris-HCl pH 6.8, 3% SDS, 10% glycerol, 1 mM EDTA, 0.004% bromophenol blue) for analysis on SDS-PAGE or samples were subjected to limited proteolysis.

### Limited proteolysis

Folding of CFTR and NBD1 was assayed through protease susceptibility. Immediately after *in vitro* translation, proteins were subjected to increasing concentrations of TPCK-trypsin (Sigma) or Proteinase K (Roche) as described before [24]. Experiments were performed using different batches of Proteinase K at different storage conditions, which resulted in minor variations in proteolytic patterns between experiments. For each experimental setup, a range of protease concentrations was used and proteolytic patterns representative of at least 3 experiments were shown.

### Lane quantitation

Exposed films containing corresponding proteolytic patterns loaded side by side in 12% SDS-PA gel, were scanned using a GS710 densitometer (Biorad). Total lane quantitation was done to determine relative intensities of protease resistant fragments using Quantity One software (Biorad).

### Temperature melts of isolated NBD1

Thermal denaturation was measured by monitoring Turbidity (aggregation) at 300 nm of 5 µM NBD1-CFTR in Buffer M (50 mM Tris-HCl, 150 mM NaCl, 5 mM MgCl_2_, 2 mM ATP, pH 7.6). Turbidity was measured every 0.5°C, rate of temperature increase was 0.5°C/min. Melting Temperature (T_M_) was determined by taking the second derivative.
